# Biographical

**Published:** 1892-09

**Authors:** 


### Biographical.

Died—Dr. Daniel W. Roudebush, at his home in Covington, Ky., after an illness of six months, August 26th, 1892.

He was born August 6th, 1821, at Goshen, Clermont Co., Ohio. He secured an education chiefly by his own effort, working during the summer and attending school during the winter. He read medicine in the office of his cousin Dr. Isaac Thecker in his native town. He afterwards studied and took up dentistry for his life work. Preparatory to this he was a pupil under the late Dr. John Allen, and also the late Dr. D. B. Wheeler, both of Cincinnati. He attended lectures at the Ohio College of Dental Surgery in 1855. In that year he opened an office in Covington where he was in active practice for thirty-seven years, except for a brief period during which he was associated professionally with Dr. J. Taft, in Cincinnati.

He was married in October, 1845, to Mrs. Nancy A. Flanders, who with one son, Dr. C. M. Roudebush, and two daughters, Mrs. C. E. Wasson and Mrs. Geo. O. Shivers, survive him.

Dr. Roudebush was passionately devoted to his profession and followed it with a most persevering industry, and to this, undoubtedly, was due, in a great degree, the failure of his health.

He was interested in whatever promoted his profession. He was quite a favorite with all who knew him, and only those could appreciate his real character.

